# MIBG scans in patients with stage 4 neuroblastoma reveal two metastatic patterns, one is associated with MYCN amplification and in MYCN-amplified tumours correlates with a better prognosis

**DOI:** 10.1007/s00259-014-2909-1

**Published:** 2014-09-30

**Authors:** Gitta Bleeker, Berthe L. van Eck-Smit, Koos H. Zwinderman, Rogier Versteeg, Max M. van Noesel, Boen L. Kam, Gertjan J. Kaspers, Annelies van Schie, Susan G. Kreissman, Gregory Yanik, Barbara Hero, Matthias Schmidt, Geneviève Laureys, Bieke Lambert, Ingrid Øra, Johannes H. Schulte, Huib N. Caron, Godelieve A. Tytgat

**Affiliations:** 1 Department of Paediatric Oncology, Academic Medical Centre/Emma Children’s Hospital, PO Box 22700, 1100 DE Amsterdam, The Netherlands; 2 Department of Oncogenomics, Academic Medical Centre, Amsterdam, Netherlands; 3 Department of Nuclear Medicine, Academic Medical Centre, Amsterdam, Netherlands; 4 Department of Biostatistics, Academic Medical Centre, Amsterdam, Netherlands; 5 Department of Paediatric Oncology/Haematology, Erasmus Medical Centre/Sophia Children’s Hospital, Rotterdam, Netherlands; 6 Department of Nuclear Medicine, Erasmus Medical Centre, Rotterdam, Netherlands; 7 Department of Paediatric Oncology, VU University Medical Centre, Amsterdam, Netherlands; 8 Department of Nuclear Medicine, VU University Medical Centre, Amsterdam, Netherlands; 9 Duke University Medical Centre, Durham, NC USA; 10 Children’s Oncology Group (COG), University of Florida, Gainesville, FL USA; 11 Department of Paediatrics, Division of Haematology and Oncology, University of Michigan, Ann Arbor, MI USA; 12 Children’s Hospital, University Hospital of Cologne, Cologne, Germany; 13 Department of Nuclear Medicine, University Hospital of Cologne, Cologne, Germany; 14 Department of Paediatric Haematology and Oncology, Ghent University Hospital, Ghent, Belgium; 15 Department of Nuclear Medicine, Ghent University Hospital, Ghent, Belgium; 16 Department of Paediatric Oncology, Lund University Hospital, Lund, Sweden; 17 University Children’s Hospital Essen, Essen, Germany; 18 Dutch Childhood Oncology Group (DCOG), The Hague, Netherlands

**Keywords:** Neuroblastoma, MIBG scan, Metastatic patterns, Metastases, Outcome

## Abstract

**Purpose:**

The aim of this study was to find clinically relevant MIBG-avid metastatic patterns in patients with newly diagnosed stage 4 neuroblastoma.

**Methods:**

Diagnostic ^123^I-MIBG scans from 249 patients (123 from a European and 126 from the COG cohort) were assessed for metastatic spread in 14 body segments and the form of the lesions: “focal” (clear margins distinguishable from adjacent background) or “diffuse” (indistinct margins, dispersed throughout the body segment). The total numbers of diffuse and focal lesions were recorded. Patients were then categorized as having lesions exclusively focal, lesions more focal than diffuse, lesions more diffuse than focal, or lesions exclusively diffuse.

**Results:**

Diffuse lesions affected a median of seven body segments and focal lesions a median of two body segments (*P* < 0.001, both cohorts). Patients with a focal pattern had a median of 2 affected body segments and those with a diffuse pattern a median of 11 affected body segments (*P* < 0.001, both cohorts). Thus, two MIBG-avid metastatic patterns emerged: “limited-focal” and “extensive-diffuse”. The median numbers of affected body segments in MYCN-amplified (MNA) tumours were 5 (European cohort) and 4 (COG cohort) compared to 9 and 11, respectively, in single-copy MYCN (MYCNsc) tumours (*P* < 0.001). Patients with exclusively focal metastases were more likely to have a MNA tumour (60 % and 70 %, respectively) than patients with the other types of metastases (23 % and 28 %, respectively; *P* < 0.001). In a multivariate Cox regression analysis, focal metastases were associated with a better event-free and overall survival than the other types of metastases in patients with MNA tumours in the COG cohort (*P* < 0.01).

**Conclusion:**

Two metastatic patterns were found: a “limited and focal” pattern found mainly in patients with MNA neuroblastoma that correlated with prognosis, and an “extensive and diffuse” pattern found mainly in patients with MYCNsc neuroblastoma.

**Electronic supplementary material:**

The online version of this article (doi:10.1007/s00259-014-2909-1) contains supplementary material, which is available to authorized users.

## Introduction

In about 90 % of patients with neuroblastoma, ^123^I-MIBG scintigraphy reveals both the primary tumour and, in stage 4 neuroblastoma, especially osteomedullary metastases [1]. Two standardized methods for scoring MIBG scans have been described: the Curie method and the SIOPEN method [2–7]. These are semiquantitative and are used for assessment of tumour load and for response evaluation. However, no detailed analyses of MIBG-avid metastatic patterns in stage 4 neuroblastoma have been reported. Moreover, as well as the number of lesions (tumour load) on ^123^I-MIBG scans at diagnosis, osteomedullary lesions can present as focal lesions, diffuse lesions or both types. So we wondered if these would represent different types of biological lesion.

As stage 4 neuroblastoma is a heterogeneous disease with varying outcomes, we hypothesized that different disease patterns exist in patients with stage 4 neuroblastoma. Therefore, the aim of this study was to find clinically relevant MIBG-avid metastatic patterns in patients with newly diagnosed stage 4 neuroblastoma.

## Materials and methods

Diagnostic ^123^I-MIBG scans from 249 patients with histologically proven [8] stage 4 neuroblastoma according to the International Neuroblastoma Staging System from two patient cohorts (the European cohort and the COG cohort) were included retrospectively. The European cohort comprised 123 patients from European collaborative centres diagnosed between October 1994 and September 2012 (Supplementary Table 1) and treated with different high-risk protocols: HR-NBL-1/SIOPEN (26 patients) [9], GPOH NB97 (21 patients) [10], NBL HR VECI (38 patients) [11], DCOG NBL 2004/GPOH (38 patients). The COG cohort comprised 126 patients from the COG A3973 high-risk protocol study diagnosed between March 2001 and March 2006 [12]. The European cohort was used as the study cohort, and the COG cohort as the validation cohort. Clinical patient data, available in the clinical trial databases of the centres and trial organizations were used. MYCN status was determined as specified in the GPOH, SIOPEN and COG trials (Southern blot, fluorescence in situ hybridization or array-based comparative genomic hybridization) [13–15].

For the European cohort, the institutional review board approved this retrospective study and the requirement to obtain informed consent was waived (European cohort) or written informed consent was obtained from all patients (or legal guardians) before study entry (COG cohort).

### ^123^I-MIBG scans

Diagnostic whole-body ^123^I-MIBG scans acquired according to protocols corresponding with European guidelines and International Neuroblastoma Risk Group taskforce recommendations were used [1, 6, 16]. Scans were excluded if metastases were non-MIBG-avid, if body parts were lacking, if the image count rate was not sufficient for adequate discrimination between body and background (inferior quality), if patients were receiving treatment with antihypertensive agents that antagonize the uptake of MIBG by neuroblastoma cells, and if no scan was available at diagnosis (Supplementary Table 2).

### Method for evaluating ^123^I-MIBG scans

**Fig. 1:**
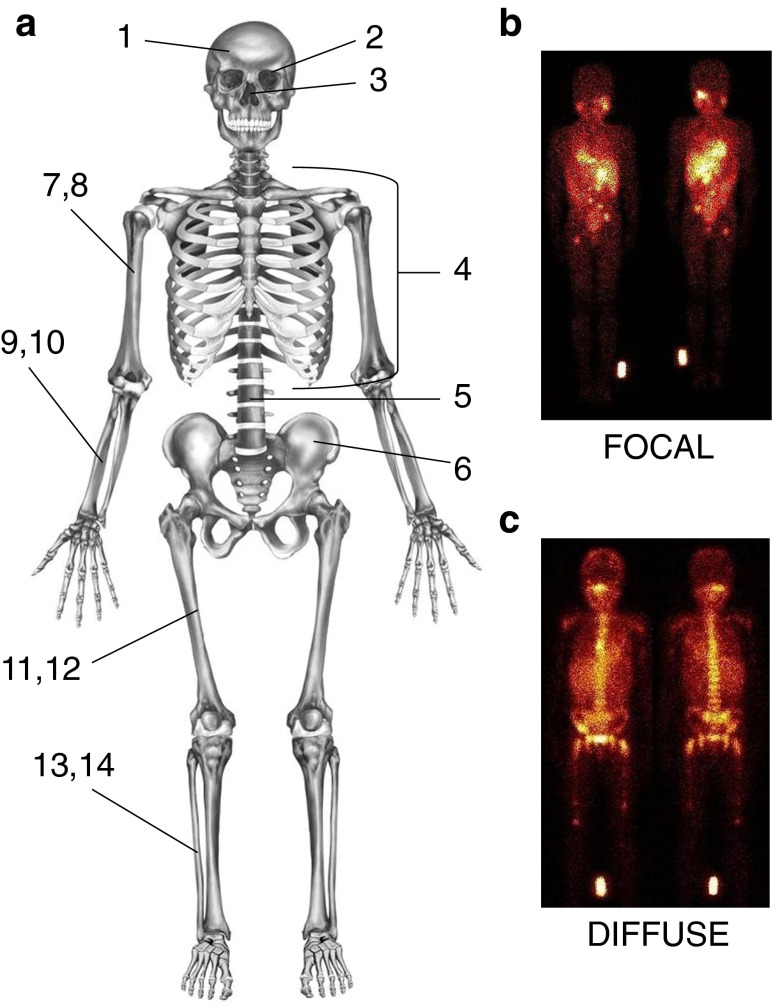
Method for evaluating MIBG-avid metastatic patterns (form and number of affected body segments). **a** Number of affected body segments: *1* dome of skull; *2* base of skull; *3* facial bones; *4* ribs, sternum, scapula and clavicles; *5* vertebral column; *6* pelvis; *7* and *8* upper arms (left and right); *9* and *10* forearms and hands (left and right); *11* and *12* upper legs (left and right); *13* and *14*: lower legs and feet (left and right). **b**, **c** Lesion form: focal (**b**) lesions imaged as hot-spots with clear margins, distinguishable from the background; diffuse (**c**) lesions without clear margins and uptake spread throughout the body segment

To be able to investigate metastatic patterns, the number of affected body segments was recorded (maximum of 14) and the form of MIBG-avid skeletal lesions was categorized as “focal” (F) or “diffuse” (D) (Fig. 1a; see Supplementary Table 3 for an example of the categorization of form). Focal metastases were hot-spots with clear margins distinguishable from the background (Fig. 1b). Diffuse metastases had no clear margins and were spread throughout the body segment (Fig. 1c). Scans were evaluated by two independent observers trained by a nuclear medicine radiologist. For the COG cohort the extension of metastases was also scored according to the Curie method [2, 6].

### Statistical analysis

Interobserver variability was quantified using the kappa coefficient before discordant findings were resolved by consensus. All reported correlations and outcome analyses were performed on the consensus scores. Correlations were tested for statistical significance using the Mann-Whitney *U* test, the Kruskal Wallis test or Fisher's Exact test. Survival was analysed using the Kaplan-Meier life-table method for the COG cohort because this cohort was homogeneously treated over a fixed time period. The median follow-up was 6.1 years (1.3 to 9.5 years). Event-free survival (EFS) was calculated as the time from diagnosis to the first event (relapse, progression, death) or last examination if no event occurred. Overall survival (OS) was calculated as the time from diagnosis to death or last examination. Differences in OS and EFS between patients with different metastatic patterns were analysed using the log-rank test. A multivariate Cox regression analysis of the form of metastases, age, and Curie score, stratified by MYCN status, was also performed. Therefore metastases were divided into two categories (dichotomized) according to form (exclusively focal versus the others, i.e. focal≥diffuse + diffuse>focal+diffuse), and the patients were divided according to the cut-off age of 18 months [17] and according to the median Curie score of 12.

To test the effect of tumour burden at diagnosis on outcome, the Curie score was determined in the COG cohort, and outcome was evaluated in term of both previously published cut-off scores of 2 and 9 [3, 7] and also a cut-off score of 12 (median score in the COG cohort).

## Results

### Patient characteristics

We included 123 out of 149 of the available diagnostic ^123^I-MIBG scans from patients of the European cohort. In the COG A3973 high-risk cohort, in 306 patients, 91 diagnostic scans were ^131^I-MIBG scans. Clinical and biological characteristics of 170 of 215 patients with ^123^I-MIBG scans were available and evaluated [12]. Of these 170 ^123^I-MIBG scans, 126 were included. Excluded scans are described in Supplementary Table 2 for the two cohorts: non-MIBG-avid metastases in 21 and 20 patients, inferior quality in 2 and 23, use of antihypertensive agents in 2 and 0, and treatment before the scanning procedure in 1 and 1 patient. The distribution of age and MYCN status was comparable in both cohorts (Table 1), except exclusion of patients younger than 12 months with single-copy MYCN (MYCNsc) neuroblastoma in the high-risk COG cohort. The characteristics of the excluded patients were comparable in the two cohorts.

**Table 1:** Patient characteristics

MYCN status	Age	European cohort (*n* = 123)^a^	COG cohort (*n* = 126)^b^
Amplified^c^	<12 months	3	2
	12–18 months	7	9
	18 months to 12 years	28	34
	≥12 years	0	0
	Unknown	1	0
	Total	39	45
Single copy	<12 months	16	0
	12–18 months	6	4
	18 months to 12 years	54	71
	≥12 years	2	5
	Unknown	0	0
	Total	78	80
No data	<12 months	0	0
	12–18 months	1	0
	18 months to 12 years	5	0
	≥12 years	0	0
	Unknown	0	1
	Total	6	1

^a^Median age 2.7 years (range 0–16.5 years)

^b^Median age 2.9 years (range 0.8–15.2 years)

^c^MYCN amplification was considered present if eight or more copies were detected [10–12]

### Focal and diffuse lesions

A total 966 lesions were identified in the European cohort (123 patients; Table 2) affecting 928 body segments. Of these lesions, 292 (30 %) were focal and 674 (70 %) were diffuse. Representative ^123^I-MIBG scans are shown in Fig. 1b, c. The 292 focal lesions were present in 101 of 123 patients, with a median of 2 per patient compared to 674 diffuse lesions in 95 of 123 patients with a median of 7 (*P* < 0.0001; Table 2). In the COG cohort A total of 984 lesions were identified in the COG cohort (123 patients) affecting 969 body segments. Of these lesions, 264 (27 %) in 95 patients were focal and 720 (73 %) in 103 patients were diffuse. Diffuse lesions affected a median of 7 body segments per patient and focal lesions a median of 2 body segments per patient (*P* < 0.0001; Table 2).

**Table 2:** Characteristics of metastatic lesions

		Focal	Diffuse	Total	*P* value
European cohort	Number of lesions	292 (30 %)	674 (70 %)	966	
	Number of patients	101	95	123	
	Lesions per patient, median (range)	2 (1–9)	7 (1–14)		<0.001
COG cohort	Number of lesions	264 (27 %)	720 (73 %)	984	
	Number of patients	95	103	126	
	Lesions per patient, median (range)	2 (1–8)	7 (1–14)		<0.001

### “Limited-focal” and “extensive-diffuse” MIBG-avid metastatic patterns

**Fig. 2:**
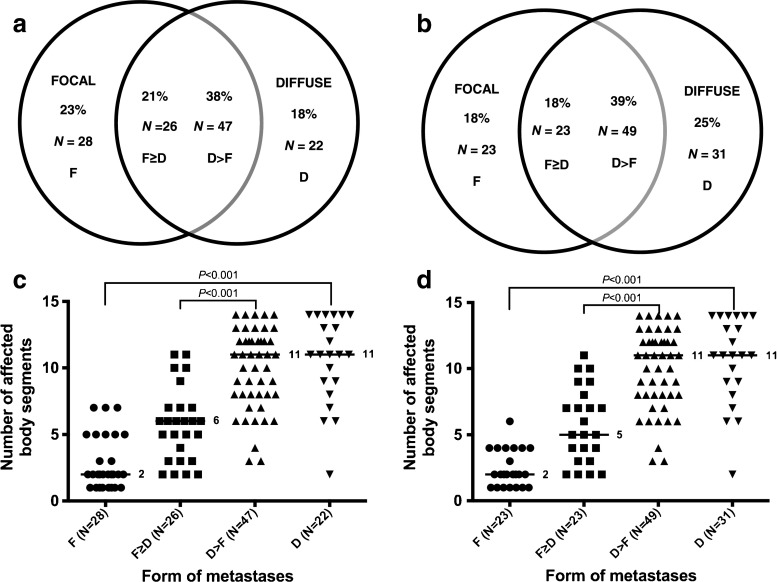
Forms of metastases and numbers of affected body segments per patient. **a**, **b** Distribution of forms of metastases in the European cohort (**a**) and the COG cohort (**b**). The numbers of patients with focal, diffuse and both types of lesions are shown. Patients were categorized as exclusively focal (*F*), exclusively diffuse (*D*), focal≥diffuse (*F≥D*) or diffuse>focal (*D>F*). **c**, **d** Relationship between form of metastases and number of affected body segments per individual patient in the European cohort (**c**) and the COG cohort (**d**). Each *data point* represents a patient; the *horizontal lines* indicate the median number of affected body segments

Patients present with only focal, only diffuse, or both forms of metastases. The numbers of patients with “exclusively focal” (focal), “focal more than/equal to diffuse” (focal≥diffuse), “more diffuse than focal” (diffuse>focal), and “exclusively diffuse” (diffuse) lesions were recorded. The distributions of the patterns were very similar in the European and COG cohorts, with a slight preponderance of the diffuse>focal pattern (38 % and 39 %), with 23 % and 18 % focal, 21 % and 18 % focal≥diffuse, and 18 % and 25 % diffuse, respectively (Fig. 2a, b).

The association between the form and the number of affected body segments were then analysed for each patient. In the European cohort, focal, focal≥diffuse, diffuse>focal and diffuse lesions were identified in a median of 2, 6, 11 and 11 body segments, respectively (Fig. 2c). There were significant differences between the form groups in relation to the number of affected body segments: focal versus diffuse, focal≥diffuse versus diffuse>focal and focal versus the three other groups, all *P* < 0.001. In the COG cohort these patterns were very similar and significant relationships between the form of metastases and the number of affected body segments were also seen (Fig. 2d). The actual numbers of focal and diffuse lesions in each individual patient are shown in Supplementary Table 4.

### “Limited-focal” patterns associated with MYCN amplification

**Fig. 3:**
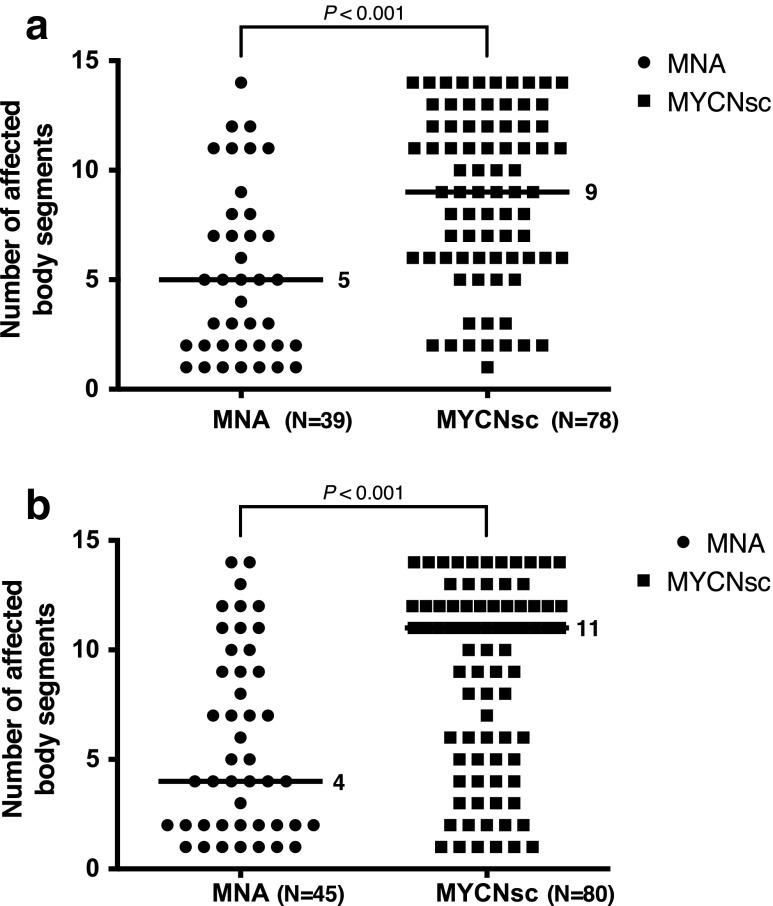
Relationship between MYCN status and number of affected body segments per patient: **a** European cohort (*n* = 123; six patients no data on MYCN status). **b** COG cohort (*n* = 126; one patient no data on MYCN status). Each *data point* represents a patient; *horizontal lines* median numbers of affected body segments. *MNA* MYCN amplification, *MYCNsc* single-copy MYCN

The differences in MIBG-avid metastatic patterns suggest a biological difference between the MYCN status groups. We therefore investigated whether the MYCN gene could be involved. MYCN status was clearly associated with the number of affected body segments (Fig. 3) as well as with the form of metastases (Table 3). Patients with MYCN-amplified (MNA) tumours had significantly fewer affected body segments. The median numbers of affected body segments in the European and COG patients with MNA tumours were 5 and 4, respectively, and in the European and COG patients with MYCNsc tumours were 9 and 11, respectively (*P* < 0.001 for both cohorts; Fig. 3). MNA tumour was found in 67 % of patients (18/27) with focal lesions in the European cohort and in 70 % of such patients (16/23) in the COG cohort (Table 3). In contrast, MNA tumour was found in only 23 % of patients (21/90) in the other metastatic groups (focal≥diffuse + diffuse>focal + diffuse; *P* < 0.001) in the European cohort and in 28 % of such patients (29/102; *P* < 0.001) in the COG cohort.

**Table 3:** MYCN status in relation to the form of metastases

MYCN status	European cohort			COG cohort		
	Form		Total	Form		Total
	Focal	All other groups^a^		Focal	All other groups^a^	
Amplified	18	21	39	16	29	45
Single copy	9	69	78	7	73	80
Total	27	90	117	23	102	125
*P* value			<0.001			<0.001

^a^Focal≥diffuse + diffuse>focal + diffuse

### Prognostic value of MIBG-avid metastatic patterns

Next, we investigated the prognostic impact of the identified MIBG-avid metastatic patterns. This analysis was only performed in the COG cohort. The European cohort received more variable treatment due to the longer inclusion period, which might have had an impact on OS and EFS, but not on the metastatic pattern as these were investigated at diagnosis only.

**Fig. 4:**
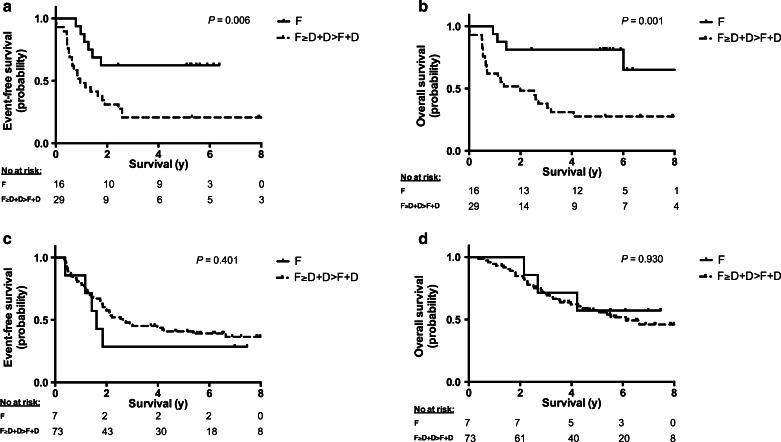
Event-free survival (EFS) and overall survival (OS) according to the form of metastases stratified by MYCN status (**a**, **b** MYCN-amplified tumours; **c**, **d** single-copy MYCN tumours). EFS (**a**, **c**) and OS (**b**, **d**) in patients with focal lesions (*F*) versus those with all other types of lesions (focal≥diffuse + diffuse>focal + diffuse, *F≥D + D>F + D*). (*No at risk* number of patients at risk, i.e. still alive, at the corresponding time points, *y* years after diagnosis)

COG patients with exclusively focal metastases had a small but statistically significant better OS, but not EFS, than patients in the other metastatic groups (95 % confidence intervals, CI: 52 ± 20 % vs. 35 ± 9 % for 5-year EFS, *P* = 0.191; 73 ± 19 % vs. 49 ± 10 % for 5-year OS, *P* = 0.050; Supplementary Fig. 1). Among patients with MNA tumours, those with focal lesions had a much better EFS and OS than those in the other metastatic groups (95 % CI: 63 ± 24 % vs. 21 ± 15 % for 5-year EFS, *P* = 0.006; 81 ± 20 % vs. 28 ± 17 % for 5-year OS, *P* = 0.001; Fig. 4a, b). Among patients with MYCNsc tumours, no significant nor large differences in EFS and OS were found between those with focal lesions and those in all other metastatic groups (95 % CI: 29 ± 34 % vs. 41 ± 12 % for 5-year EFS, *P* = 0.401; 57 ± 37 % vs. 58 ± 12 % for 5-year OS, *P* = 0.930; Fig. 4c, d).

In the separate multivariate Cox regression analyses in patients with MNA tumour and those with MYCNsc tumours the form of metastases in MNA neuroblastomas showed independent prognostic value in the presence of potential prognostic factors (age and Curie score at diagnosis; Table 4). Using the Curie score as a dichotomized variable with previously published cut-off scores of 2 and 9 [3, 7] and also with a cut-off score of 12 (median score in the COG cohort), did not result in a significant prediction of outcome in either the univariate or the multivariate analyses. So correcting for the Curie score did not have impact on outcome.

**Table 4:** Multivariate Cox regression analysis of survival in the COG cohort stratified by MYCN status

MYCN status	Variable	5-year event-free survival			5-year overall survival		
		Hazard ratio	95 % CI	*P* value	Hazard ratio	95 % CI	*P* value
Amplified	Age (<18 months vs. ≥18 months)	1.3	0.6–3.0	0.540	1.4	0.6–3.5	0.472
	Curie score (<12 vs. ≥12)	1.2	0.5–2.9	0.607	1.2	0.5–2.9	0.679
	Form of metastases (focal vs. all other forms^a^)	0.3	0.1–0.7	0.010	0.2	0.04–0.6	0.005
Single copy	Age (<18 months vs. ≥18 months)	0.9	0.2–3.7	0.860	0.5	0.07–3.8	0.497
	Curie score (<12 vs. ≥12)	0.7	0.3–1.4	0.291	0.9	0.4–1.9	0.697
	Form of metastases (focal vs. all other forms^a^)	1.9	0.7–5.6	0.230	1.1	0.3–4.3	0.848

^a^Focal≥diffuse + diffuse>focal + diffuse

We conclude that in patients with MNA tumours, patients with exclusively focal metastases have a significantly better outcome than patients with (additional) diffuse metastases.

### Interobserver variability

The interobserver variability in evaluating the ^123^I-MIBG scans was “moderate” to “almost perfect”, with median κ values for affected body segments and for form of metastases per body segment of 0.9 (0.7–1.0) and 0.5 (0.3–1.0), respectively in the European cohort, and of 0.9 (0.7–0.9) and 0.6 (0.3–0.8), respectively, in the COG cohort (for details see Supplementary Table 5). The κ value for the form of metastases on a patient basis categorized as focal vs. focal≥diffuse + diffuse>focal + diffuse was 0.7 (*P* < 0.001) with 89 % concordant findings. All discordant findings were resolved by consensus. Performing all analyses again with single observer rates did not change the findings.

## Discussion

This study identified two MIBG-avid metastatic patterns in patients with newly diagnosed stage 4 neuroblastoma: a “limited and focal” pattern found mainly in patients with MNA neuroblastoma, and an “extensive and diffuse” pattern found mainly in patients with MYCNsc neuroblastoma. The obvious difference between focal and diffuse metastases is still not understood. In international guidelines skeletal uptake of MIBG is reported to be visible as focal or diffuse lesions [18], and the SIOPEN scoring method distinguishes between discrete foci and diffuse lesions [6]. In the literature, “focal” lesions are mostly reported as bone metastases [19, 20] and “diffuse” lesions as bone marrow metastases [19, 21–24]. As almost 95 % of patients with stage 4 neuroblastoma have bone marrow involvement at diagnosis, the nature of focal and diffuse lesions was not studied in our cohort. This needs to be done in a prospective study, comparing MIBG scintigraphy, immunocytological bone marrow involvement and MRI for anatomical localization of the lesions.

To our knowledge, no classification of stage 4 tumours based on MIBG-avid metastatic patterns has been described. The recognition of two different metastatic patterns in stage 4 neuroblastoma suggests that multiple and different underlying molecular alterations might be involved in the process of metastases. Indeed the focal pattern was significantly associated with amplification of MYCN, which is considered a major tumour-driving gene. Furthermore, only in patients with MNA tumours, focal metastases were significantly associated with a more favourable outcome than diffuse metastases, and in this subset of patients different biological processes might be identified. Why this phenomenon was not seen in patients with MYCNsc tumours needs to be studied further.

A major question arising from this study is whether MYCN activity is responsible for the focal growth of metastatic lesions, and whether and how it relates to the fewer affected body segments. Not all patients with predominantly focal lesions had MNA tumours. However, in another study in our laboratory a subset of MYCNsc neuroblastomas were found to have high MYCN protein expression. We hypothesize that the patients with focal lesions with MYCNsc tumours might have had this MYCN expression profile [25]. The intriguing results of this study require substantial biological research, e.g. association with gene expression and molecular data, and in vitro and in vivo MYCN manipulation studies, to elucidate the functional role for MYCN in the metastatic spread of neuroblastoma cells. MYCN expression has been reported to correlate with a lower norepinephrine transporter (NET) protein expression, and in turn a lower NET protein expression was correlated with low MIBG avidity [26]. As we found that patients with MNA neuroblastoma had not only a predominantly focal form of metastases but also significantly fewer involved body segments on MIBG imaging at diagnosis, we hypothesize that the lower NET expression in MNA tumours causes the focal pattern.

Although two well-performing methods for scoring MIBG scans are used internationally, we developed our own method of evaluation as a tool to find MIBG-avid metastatic patterns with a qualitative variable (the form of metastases per body segment) as the key variable [2, 3, 6] (Supplementary Table 6). The novelty of our findings is that the form of metastases at diagnosis, an aspect that is not scored with the Curie method, was significantly correlated with outcome in the MNA group. If a prospective study in a homogeneously treated cohort can confirm the prognostic relevance of the metastatic pattern in patients with stage 4 neuroblastoma, eventually patients with stage 4 neuroblastoma might be subdivided in two risk groups. In addition, in future it might then be possible to treat patients with different metastatic patterns according to different treatment protocols that are more targeted at their biological background.

Although the distinction between focal and diffuse metastases was not always very clear, especially because sometimes the two forms of metastases were present in one body segment, all discordant findings could be resolved by consensus. Furthermore, the results of our correlation and prognostic analyses were comparable when using single observer scores. Since the study cohort consisted of ^123^I-MIBG scans obtained over a long period of time, scan quality might have been heterogeneous and not equally distributed between the subgroups. Therefore our results should be confirmed in a cohort of patients scanned according to a uniform protocol.

In this study we included only ^123^I-MIBG scans, but since Naranjo et al. reported no difference in outcome between scoring of ^123^I-MIBG and ^131^I-MIBG scans [12], it is debatable whether ^131^I-MIBG scans should have been excluded. However, this project was started many years ago and at that time ^123^I-MIBG scans were reported to be of better quality. In the Cox regression analysis, we included only the COG cohort, a homogeneous patient population treated according to one protocol (COG A3973 protocol). The European cohort was a heterogeneous cohort because these patients had been treated according to different protocols, and therefore this was not an ideal cohort for studying survival. A limitation of the COG cohort was that not all patients had a follow-up of 5 years. A prospective study including patients from the same treatment protocol with only digital diagnostic ^123^I-MIBG scans performed according to the same scanning procedures and evaluated by central review might resolve these problems.

In conclusion, our study clearly showed the existence of two relevant MIBG-avid metastatic patterns in newly diagnosed neuroblastoma: an “extensive and diffuse” MIBG-avid metastatic pattern found mainly in patients with MYCNsc tumours, and a “limited and focal” pattern found mainly in patients with MNA tumours. In patients with MNA tumours, focal metastases had a better prognosis than the other types of metastases. These two patterns most likely reflect different biological processes that should be explored further with the aim of providing a better understanding of the heterogeneous behaviour of high-risk tumours.

## Electronic supplementary material

Below is the link to the electronic supplementary material.

Supplementary Table 1 (DOC 31 kb)

Supplementary Table 2 (DOC 28 kb)

Supplementary Table 3 (DOC 84 kb)

Supplementary Table 4 (DOC 100 kb)

Supplementary Table 5 (DOC 101 kb)

Supplementary Table 6 (DOC 36 kb)

Supplementary Fig. 1 (JPEG 341 kb)
(JPEG 316 kb)

High Resolution Image (EPS 114 kb) High Resolution Image (EPS 110 kb)
