# Supplementary material for: MIBG scans in patients with stage 4 neuroblastoma reveal two metastatic patterns, one is associated with MYCN amplification and in MYCN-amplified tumours correlates with a better prognosis
Source: Eur J Nucl Med Mol Imaging. 2014 Sep 30;42(2):222–30. doi: 10.1007/s00259-014-2909-1 (PMC4315489; doi:10.1007/s00259-014-2909-1)
Supplement: Supplementary file 1 — (DOC 31 kb) [file 259_2014_2909_MOESM1_ESM.doc]

**Supplemental Table 1: European collaborative centres of European cohort**

| **European centre:** | **Location:** | **Number of included patients:** | **Number of excluded patients:** |
| --- | --- | --- | --- |
| Academic Medical Centre (AMC)/ Emma Children’s Hospital | Amsterdam, the Netherlands | 46 | 12 |
| Erasmus MC/ Sophia Children’s Hospital | Rotterdam, the Netherlands | 17 | 2 |
| VU University Medical Centre (VUmc) | Amsterdam, the Netherlands | 9 | 5 |
| Groningen University Medical Centre | Groningen, the Netherlands | 4 | 0 |
| University Hospital of Cologne | Cologne, Germany | 18 | 2 |
| Essen University Hospital | Essen, Germany | 3 | 0 |
| Ghent University Hospital | Ghent, Belgium | 16 | 2 |
| Lund University Hospital | Lund, Sweden | 10 | 3 |
| **TOTAL:** | **149** | **123** | **26** |

For reasons of exclusion see Supplemental Table 2.
