# Supplementary material for: MIBG scans in patients with stage 4 neuroblastoma reveal two metastatic patterns, one is associated with MYCN amplification and in MYCN-amplified tumours correlates with a better prognosis
Source: Eur J Nucl Med Mol Imaging. 2014 Sep 30;42(2):222–30. doi: 10.1007/s00259-014-2909-1 (PMC4315489; doi:10.1007/s00259-014-2909-1)
Supplement: Supplementary file 2 — (DOC 28 kb) [file 259_2014_2909_MOESM2_ESM.doc]

**Supplemental Table 2: Excluded ^123^I-MIBG scans**

|  |  | **EUROPEAN COHORT** | **COG COHORT** |
| --- | --- | --- | --- |
| **Available** |  | **149** | **170** |
| **Exclusion** | MIBG-non-avid metastases | 21 (14%) | 20 (12%) |
|  | Scan not complete / inferior quality | 2 (1%) | 23 (14%) |
|  | Anti-hypertensive agents | 2 (1%) | 0 (0%) |
|  | No scan available at diagnosis | 1 (0.6%) | 1 (0.6%) |
| **Inclusion** |  | **123 (83%)** | **126 (74%)** |
