# Supplementary material for: MIBG scans in patients with stage 4 neuroblastoma reveal two metastatic patterns, one is associated with MYCN amplification and in MYCN-amplified tumours correlates with a better prognosis
Source: Eur J Nucl Med Mol Imaging. 2014 Sep 30;42(2):222–30. doi: 10.1007/s00259-014-2909-1 (PMC4315489; doi:10.1007/s00259-014-2909-1)
Supplement: Supplementary file 3 — (DOC 84 kb) [file 259_2014_2909_MOESM3_ESM.doc]

**OVERVIEW**

| **Study number:** |  |
| --- | --- |
| **Date of scan:** |  |
| **Date of scoring:** |  |
| **First / second observer:** |  |
| **Stage:** |  |

Metastases:

| **Localization****:** | **Sk1 ^I^** | **Sk2 ^II^** | **Sk3 ^III^** | **VertC ^IV^** | **Rscs ^V^** | **Pel ^VI^** |
| --- | --- | --- | --- | --- | --- | --- |
|  |  |  |  |  |  |
| **Aspect I**^7^**:** |  |  |  |  |  |  |
| **Extension**^8^**:** |  |  |  |  |  |  |
| **Side of the body**^9^**:** |  |  |  |  |  |  |
| **Proximal or distal**^10^**:** |  |  |  |  |  |  |
| **Number^11^:** |  |  |  |  |  |  |
| **Comments:** |  |  |  |  |  |  |

| **Localization****:** | **UpA, right ^VII^** | **UpA, left^VIII^** | **FoA,**  **right ^IX^** | **FoA,**  **left^X^** |
| --- | --- | --- | --- | --- |
|  |  |  |  |
| **Aspect I**^7^**:** |  |  |  |  |
| **Extension**^8^**:** |  |  |  |  |
| **Side of the body**^9^**:** |  |  |  |  |
| **Proximal or distal**^10^**:** |  |  |  |  |
| **Number^11^:** |  |  |  |  |
| **Comments:** |  |  |  |  |

| **Localization****:** | **UpL, right^XI^** | **UpL, leftXII** | **LLF, right^XIII^** | **LLF, left ^XIV^** | **ST^XV^** |
| --- | --- | --- | --- | --- | --- |
|  |  |  |  |  |
| **Aspect I**^7^**:** |  |  |  |  |  |
| **Extension**^8^**:** |  |  |  |  |  |
| **Side of the body**^9^**:** |  |  |  |  |  |
| **Proximal or distal**^10^**:** |  |  |  |  |  |
| **Number^11^:** |  |  |  |  |  |
| **Comments:** |  |  |  |  |  |

**EXPLANATION**

Terms:

| Localization: | Tick where appropriate. |
| --- | --- |
| Alignment: | 1: sharp, 2: blurred. |
| Distribution: | 1: homogenous, 2: heterogeneous uptake. |
| Intensity: | 0: no uptake, 1: dubious uptake, 2: obvious uptake (< physiologic liver uptake), 3: strong uptake (>physiologic liver uptake). |
| Cold region: | Negative spot (related to necrosis or bleeding)? 1: yes, 2: no. |
| MIBG-outcome: | MIBG uptake? 1: yes (positive), 2: no (negative). |
| Aspect I: | 1: focal, 2: diffuse; 3: both. |
| Extension: | 0: no uptake.  If circumscript: 1. and number of lesions.  If diffuse: 2: more than one lesion (<50% of segment), 3: massive involvement (>50% of segment). |
| Side of the body: | 1: Left, 2: right, 3: left > right, 4: right > left, 5: left = right. |
| Proximal or distal: | For limbs only. 1: proximal, 2: central, 3: distal, 4: spread over the whole compartment, 5: from proximal towards distal. |
| Number | If possible, count the number of distinct metastases in the body segment. |

Abbreviations:

|  | Dome of skull. |
| --- | --- |
|  | Base of skull. |
|  | Facial bones. |
|  | Vertebral column. |
|  | Ribs, sternum, clavicle, scapula. |
|  | Pelvis. |
|  | Upper arms, right. |
|  | Upper arms, left. |
|  | Fore arms and hands, right. |
|  | Fore arms and hands, left. |
|  | Upper legs, right. |
|  | Upper legs, left. |
|  | Lower legs and feet, right. |
|  | Lower legs and feet, left |
|  | Soft tissue: liver, lymph nodes, other. |
