# Supplementary material for: MIBG scans in patients with stage 4 neuroblastoma reveal two metastatic patterns, one is associated with MYCN amplification and in MYCN-amplified tumours correlates with a better prognosis
Source: Eur J Nucl Med Mol Imaging. 2014 Sep 30;42(2):222–30. doi: 10.1007/s00259-014-2909-1 (PMC4315489; doi:10.1007/s00259-014-2909-1)
Supplement: Supplementary file 4 — (DOC 100 kb) [file 259_2014_2909_MOESM4_ESM.doc]

**Supplemental Table 4: Number of focal and diffuse lesions per patient**

**A: European cohort**

|  |  | **DIFFUSE LESIONS** | | | | | | | | | | | | | | |  |
| --- | --- | --- | --- | --- | --- | --- | --- | --- | --- | --- | --- | --- | --- | --- | --- | --- | --- |
|  |  | ***0*** | ***1*** | ***2*** | ***3*** | ***4*** | ***5*** | ***6*** | ***7*** | ***8*** | ***9*** | ***10*** | ***11*** | ***12*** | ***13*** | ***14*** | ***TOTAL*** |
| **FOCAL LESIONS** | ***0*** | 0 | 0 | 1 | 0 | 0 | 0 | 2 | 1 | 2 | 1 | 1 | 5 | 2 | 1 | 6 | 22 |
|  | ***1*** | 11 | 6 | 2 | 1 | 0 | 2 | 2 | 4 | 2 | 0 | 1 | 0 | 1 | 1 | 0 | 33 |
|  | ***2*** | 7 | 1 | 1 | 1 | 1 | 0 | 1 | 1 | 1 | 3 | 2 | 1 | 2 | 1 | 1 | 24 |
|  | ***3*** | 2 | 0 | 1 | 1 | 1 | 0 | 0 | 1 | 0 | 1 | 1 | 0 | 0 | 0 | 1 | 9 |
|  | ***4*** | 0 | 2 | 2 | 2 | 0 | 1 | 0 | 1 | 0 | 1 | 1 | 0 | 0 | 0 | 0 | 10 |
|  | ***5*** | 5 | 1 | 0 | 0 | 0 | 1 | 1 | 1 | 2 | 0 | 0 | 1 | 1 | 0 | 0 | 13 |
|  | ***6*** | 3 | 1 | 2 | 1 | 0 | 1 | 0 | 0 | 0 | 0 | 0 | 0 | 0 | 0 | 0 | 8 |
|  | ***7*** | 0 | 0 | 0 | 0 | 0 | 1 | 0 | 1 | 0 | 0 | 0 | 0 | 0 | 0 | 0 | 2 |
|  | ***8*** | 0 | 0 | 0 | 0 | 0 | 0 | 0 | 0 | 0 | 1 | 0 | 0 | 0 | 0 | 0 | 1 |
|  | ***9*** | 0 | 0 | 0 | 0 | 1 | 0 | 0 | 0 | 0 | 0 | 0 | 0 | 0 | 0 | 0 | 1 |
|  | ***10*** | 0 | 0 | 0 | 0 | 0 | 0 | 0 | 0 | 0 | 0 | 0 | 0 | 0 | 0 | 0 | 0 |
|  | ***11*** | 0 | 0 | 0 | 0 | 0 | 0 | 0 | 0 | 0 | 0 | 0 | 0 | 0 | 0 | 0 | 0 |
|  | ***12*** | 0 | 0 | 0 | 0 | 0 | 0 | 0 | 0 | 0 | 0 | 0 | 0 | 0 | 0 | 0 | 0 |
|  | ***13*** | 0 | 0 | 0 | 0 | 0 | 0 | 0 | 0 | 0 | 0 | 0 | 0 | 0 | 0 | 0 | 0 |
|  | ***14*** | 0 | 0 | 0 | 0 | 0 | 0 | 0 | 0 | 0 | 0 | 0 | 0 | 0 | 0 | 0 | 0 |
|  | ***TOTAL*** | 28 | 11 | 9 | 6 | 3 | 6 | 6 | 10 | 7 | 7 | 6 | 7 | 6 | 3 | 8 | 123 |

**B: COG cohort**

|  |  | **DIFFUSE LESIONS** | | | | | | | | | | | | | | |  |
| --- | --- | --- | --- | --- | --- | --- | --- | --- | --- | --- | --- | --- | --- | --- | --- | --- | --- |
|  |  | ***0*** | ***1*** | ***2*** | ***3*** | ***4*** | ***5*** | ***6*** | ***7*** | ***8*** | ***9*** | ***10*** | ***11*** | ***12*** | ***13*** | ***14*** | ***TOTAL*** |
| **FOCAL LESIONS** | ***0*** | 0 | 6 | 2 | 1 | 1 | 1 | 2 | 0 | 0 | 0 | 2 | 3 | 4 | 4 | 5 | 31 |
|  | ***1*** | 7 | 4 | 1 | 0 | 1 | 1 | 0 | 0 | 0 | 0 | 1 | 2 | 1 | 3 | 1 | 22 |
|  | ***2*** | 8 | 3 | 1 | 1 | 1 | 0 | 2 | 3 | 0 | 7 | 5 | 0 | 2 | 0 | 0 | 33 |
|  | ***3*** | 1 | 1 | 1 | 0 | 1 | 0 | 0 | 1 | 1 | 3 | 1 | 0 | 1 | 0 | 0 | 11 |
|  | ***4*** | 6 | 2 | 0 | 1 | 0 | 0 | 1 | 1 | 1 | 1 | 1 | 1 | 0 | 0 | 0 | 15 |
|  | ***5*** | 0 | 1 | 2 | 0 | 1 | 0 | 2 | 0 | 1 | 0 | 0 | 0 | 0 | 0 | 0 | 7 |
|  | ***6*** | 1 | 1 | 0 | 1 | 0 | 0 | 0 | 0 | 0 | 0 | 0 | 0 | 0 | 0 | 0 | 3 |
|  | ***7*** | 0 | 1 | 0 | 0 | 1 | 0 | 0 | 0 | 0 | 0 | 0 | 0 | 0 | 0 | 0 | 2 |
|  | ***8*** | 0 | 0 | 2 | 0 | 0 | 0 | 0 | 0 | 0 | 0 | 0 | 0 | 0 | 0 | 0 | 2 |
|  | ***9*** | 0 | 0 | 0 | 0 | 0 | 0 | 0 | 0 | 0 | 0 | 0 | 0 | 0 | 0 | 0 | 0 |
|  | ***10*** | 0 | 0 | 0 | 0 | 0 | 0 | 0 | 0 | 0 | 0 | 0 | 0 | 0 | 0 | 0 | 0 |
|  | ***11*** | 0 | 0 | 0 | 0 | 0 | 0 | 0 | 0 | 0 | 0 | 0 | 0 | 0 | 0 | 0 | 0 |
|  | ***12*** | 0 | 0 | 0 | 0 | 0 | 0 | 0 | 0 | 0 | 0 | 0 | 0 | 0 | 0 | 0 | 0 |
|  | ***13*** | 0 | 0 | 0 | 0 | 0 | 0 | 0 | 0 | 0 | 0 | 0 | 0 | 0 | 0 | 0 | 0 |
|  | ***14*** | 0 | 0 | 0 | 0 | 0 | 0 | 0 | 0 | 0 | 0 | 0 | 0 | 0 | 0 | 0 | 0 |
|  | ***TOTAL*** | 23 | 19 | 9 | 4 | 6 | 2 | 7 | 5 | 3 | 11 | 10 | 6 | 8 | 7 | 6 | 126 |

| F: |  |
| --- | --- |
| F≥D: |  |
| D>F: |  |
| D: |  |

Legends:

The number of focal and diffuse lesions per patient is shown on the two axes; the number of patients is shown in the table for the combined numbers of focal and diffuse lesions. E.g. 3 patients had 9 diffuse (x-axis) and 2 focal lesions (y-axis). These were coloured blue, because this represents diffuse>focal lesions.

A: European cohort (*N* = 123).

B: COG cohort (*N* = 126).

Abbreviations:

The colours represent the patient groups. F: exclusively focal lesions, F≥D: focal≥diffuse lesions, D>F: diffuse>focal lesions, D: exlusively diffuse lesions.
