# Supplementary material for: MIBG scans in patients with stage 4 neuroblastoma reveal two metastatic patterns, one is associated with MYCN amplification and in MYCN-amplified tumours correlates with a better prognosis
Source: Eur J Nucl Med Mol Imaging. 2014 Sep 30;42(2):222–30. doi: 10.1007/s00259-014-2909-1 (PMC4315489; doi:10.1007/s00259-014-2909-1)
Supplement: Supplementary file 5 — (DOC 101 kb) [file 259_2014_2909_MOESM5_ESM.doc]

**Supplemental Table 5: Inter-observer variability**

**A: European cohort**

| **Body-segment:** | **Variable:** | **Kappa** | ***P*** | ***N*** | **% concordant** |
| --- | --- | --- | --- | --- | --- |
| Dome of skull | Metastasis | 0.9 | 0.000 | 109 | 96% |
|  | Form | 0.7 | 0.000 | 47 | 87% |
| Base of skull | Metastasis | 0.7 | 0.000 | 109 | 84% |
|  | Form | 0.5 | 0.000 | 31 | 94% |
| Facial bones and orbits | Metastasis | 0.7 | 0.000 | 109 | 85% |
|  | Form | 0.4 | 0.019 | 25 | 88% |
| Vertebral column | Metastasis | 0.9 | 0.000 | 109 | 95% |
|  | Form | 0.3 | 0.005 | 38 | 79% |
| Thoracic cage | Metastasis | 0.7 | 0.000 | 109 | 85% |
|  | Form | 0.6 | 0.000 | 35 | 77% |
| Pelvis | Metastasis | 0.8 | 0.000 | 109 | 91% |
|  | Form | 0.4 | 0.000 | 57 | 67% |
| Upper arms, right | Metastasis | 0.9 | 0.000 | 109 | 96% |
|  | Form | 0.6 | 0.000 | 45 | 80% |
| Upper arms, left | Metastasis | 0.9 | 0.000 | 109 | 94% |
|  | Form | 0.6 | 0.000 | 38 | 79% |
| Fore arms and hands, right | Metastasis | 0.9 | 0.000 | 109 | 96% |
|  | Form | 0.4 | 0.134 | 9 | 78% |
| Fore arms and hands, left | Metastasis | 0.9 | 0.000 | 109 | 98% |
|  | Form | * | * | 10 | 80% |
| Upper legs, right | Metastasis | 0.8 | 0.000 | 109 | 95% |
|  | Form | 0.7 | 0.000 | 69 | 81% |
| Upper legs, left | Metastasis | 0.8 | 0.000 | 109 | 94% |
|  | Form | 0.6 | 0.000 | 66 | 80% |
| Lower legs and feet, right | Metastasis | 0.9 | 0.000 | 109 | 93% |
|  | Form | 0.7 | 0.000 | 43 | 84% |
| Lower legs and feet, left | Metastasis | 0.8 | 0.000 | 109 | 90% |
|  | Form | 0.8 | 0.000 | 40 | 88% |

**B: COG cohort**

| **Body-segment:** | **Variable:** | **Kappa** | ***P*** | ***N*** | **% Concordant** |
| --- | --- | --- | --- | --- | --- |
| Dome of skull | Metastasis | 0.9 | 0.000 | 126 | 97% |
|  | Form | 0.6 | 0.000 | 71 | 83% |
| Base of skull | Metastasis | 0.7 | 0.000 | 126 | 87% |
|  | Form | * | * | 54 | 81% |
| Facial bones and orbits | Metastasis | 0.7 | 0.000 | 126 | 85% |
|  | Form | 0.3 | 0.006 | 44 | 73% |
| Vertebral column | Metastasis | 0.9 | 0.000 | 126 | 93% |
|  | Form | 1.0 | 0.000 | 57 | 100% |
| Thoracic cage | Metastasis | 0.8 | 0.000 | 126 | 88% |
|  | Form | 0.4 | 0.000 | 61 | 71% |
| Pelvis | Metastasis | 0.9 | 0.000 | 126 | 96% |
|  | Form | 0.4 | 0.000 | 87 | 72% |
| Upper arms, right | Metastasis | 1.0 | 0.000 | 126 | 99% |
|  | Form | 0.5 | 0.000 | 80 | 75% |
| Upper arms, left | Metastasis | 0.9 | 0.000 | 126 | 94% |
|  | Form | 0.5 | 0.000 | 63 | 78% |
| Fore arms and hands, right | Metastasis | 0.8 | 0.000 | 126 | 94% |
|  | Form | 0.4 | 0.347 | 17 | 76% |
| Fore arms and hands, left | Metastasis | 0.9 | 0.000 | 126 | 98% |
|  | Form | 0.7 | 0.002 | 18 | 89% |
| Upper legs, right | Metastasis | 0.9 | 0.000 | 126 | 97% |
|  | Form | 0.6 | 0.000 | 98 | 80% |
| Upper legs, left | Metastasis | 0.9 | 0.000 | 126 | 95% |
|  | Form | 0.5 | 0.000 | 87 | 76% |
| Lower legs and feet, right | Metastasis | 0.9 | 0.000 | 126 | 94% |
|  | Form | 0.6 | 0.000 | 70 | 83% |
| Lower legs and feet, left | Metastasis | 0.9 | 0.000 | 126 | 95% |
|  | Form | 0.6 | 0.000 | 69 | 83% |

*: No kappa statistic was possible for the form of metastases in this body-segment, because one of the two observers assigned equal scores (all diffuse) concerning the form of this body-segment for the 10 patients with metastases in this body-segment, so no comparison was possible.

Variables:

1. Metastasis: body-segment affected with metastases (yes/no)?
2. Form: focal or diffuse lesions in body-segments that were affected. So the number (*N*) might be lower than for metastases, because only those body-segments that were affected could be scored for the form.

Abbreviations:

Kappa: kappa statistic, *N*: number of patients in kappa analysis.

A: European cohort

*N*: 109 of 126 MIBG scans in the European cohort were evaluated by two independent observers, 17 MIBG scans by one.

B: COG cohort
