# Supplementary material for: MIBG scans in patients with stage 4 neuroblastoma reveal two metastatic patterns, one is associated with MYCN amplification and in MYCN-amplified tumours correlates with a better prognosis
Source: Eur J Nucl Med Mol Imaging. 2014 Sep 30;42(2):222–30. doi: 10.1007/s00259-014-2909-1 (PMC4315489; doi:10.1007/s00259-014-2909-1)
Supplement: Supplementary file 6 — (DOC 36 kb) [file 259_2014_2909_MOESM6_ESM.doc]

**Supplemental Table 6: Overview of MIBG scoring methods**

|  | **Curie**  **semi-quantitative** | **SIOPEN**  **semi-quantitative / qualitative** | **Amsterdam**  **qualitative** | **DIFFERENCES** |
| --- | --- | --- | --- | --- |
| **Number of skeletal body segments** | 9   - head and face - neck and back vertebral column - ribs and sternum - lumbar and sacral column - pelvis - arms - fore arms and hands - thighs - legs and feet | 12   - skull - thoracic cage - proximal right upper limb - distal right upper limb - proximal left upper limb - distal left upper limb - spine - pelvis - proximal right - lower limb - distal right lower limb - proximal left lower limb - distal left lower limb | 14   - dome of skull - base of skull - facial bones and orbits - vertebral column - thoracic cage - pelvis - proximal right upper limb - distal right upper limb - proximal left upper limb - distal left upper limb - proximal right - lower limb - distal right lower limb - proximal left lower limb - distal left lower limb | Amsterdam *v* Curie:   - 5 body segments extra. - Head and face divided in three body segments (i.e. dome of skull, base of skull, facial bones+orbits) - Vertebral column scored as one entity instead of two. - Limbs divided in left and right limbs.   Amsterdam *v* SIOPEN:   - Two body segments extra. - Skull divided in three body segments. |
| **Soft tissue** | Yes | Yes | No | Amsterdam scored only skeletal segments and no soft tissue. |
| **Quantitative score** | Extension score (0-30):  0: no sites per segment;  1: one site per segment;  2: more than one site per segment;  3: diffuse involvement (**>**50% of the segment).  Intensity score (0-30):  0: no uptake;  1: doubtful uptake;  2: definite uptake less than liver;  3: intense uptake greater than that of liver | Extension score (0-72):  0: no involvement;  1: one discrete lesion;  2: two discrete lesions;  3: three discrete lesions;  4: 3 discrete foci or a single diffuse lesion involving **<**50% of a bone;  5: diffuse involvement of ≥50% to 95% of whole bone;  6: diffuse involvement of the entire bone. | Number of affected body segments (0-14) | Amsterdam did not score extension within body segments.  Amsterdam did not score intensity within body segments. |
| **Qualitative score** | No | Discrete foci *v* diffuse lesions. | Focal *v* diffuse lesions. | The Curie-method does not include qualitative scoring.  The SIOPEN- and Amsterdam-method both include qualitative aspect. |

Abbreviations:

SIOPEN: European SIOP Neuroblastoma Group; SIOP: International Society of Paediatric Oncology.
