# Supplementary figures and images for: MIBG scans in patients with stage 4 neuroblastoma reveal two metastatic patterns, one is associated with MYCN amplification and in MYCN-amplified tumours correlates with a better prognosis
Source: Eur J Nucl Med Mol Imaging. 2014 Sep 30;42(2):222–30. doi: 10.1007/s00259-014-2909-1 (PMC4315489; doi:10.1007/s00259-014-2909-1)

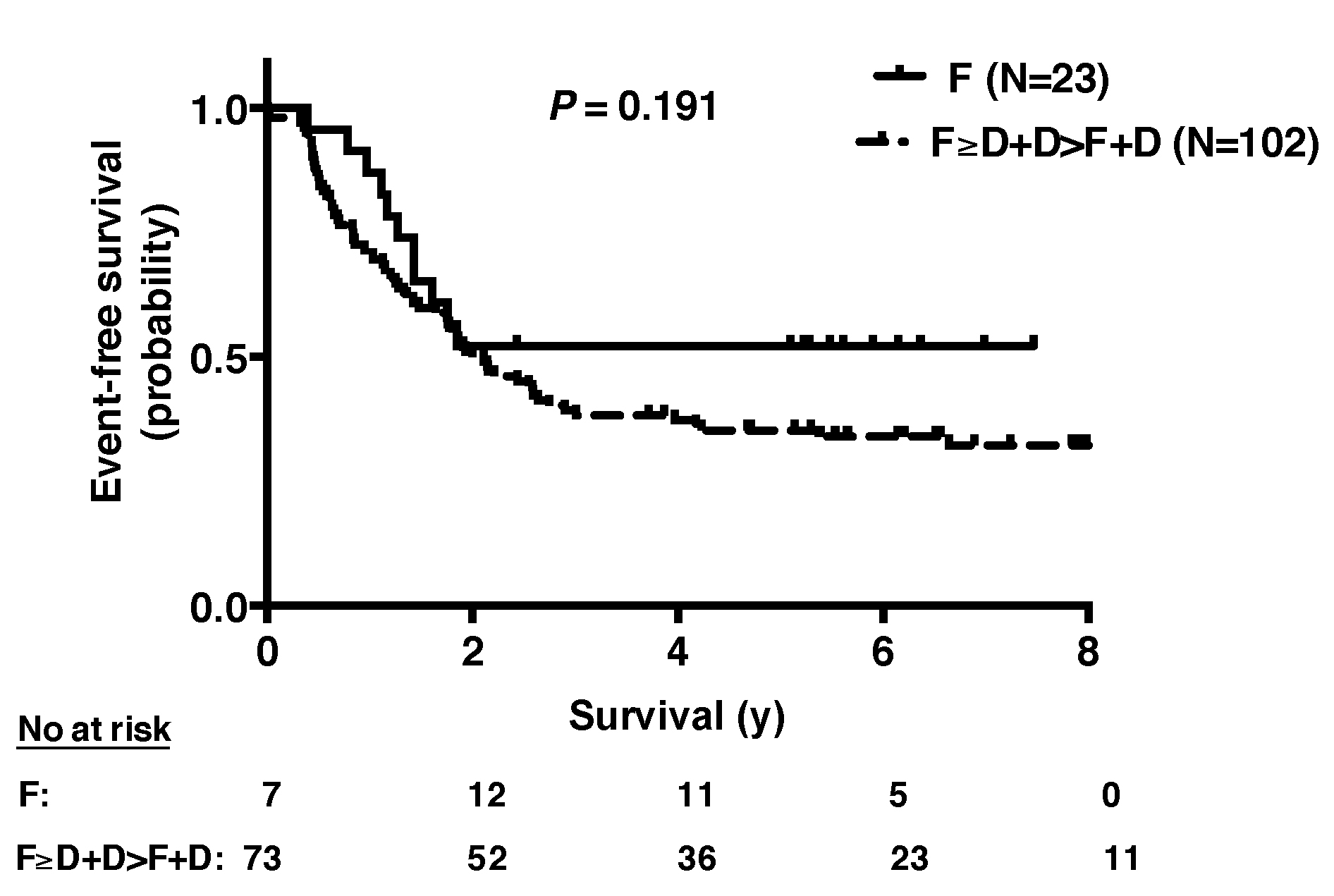

Supplement: Supplementary Fig. 1 — (JPEG 341 kb) (JPEG 316 kb) [file 259_2014_2909_Fig5_ESM.jpg]

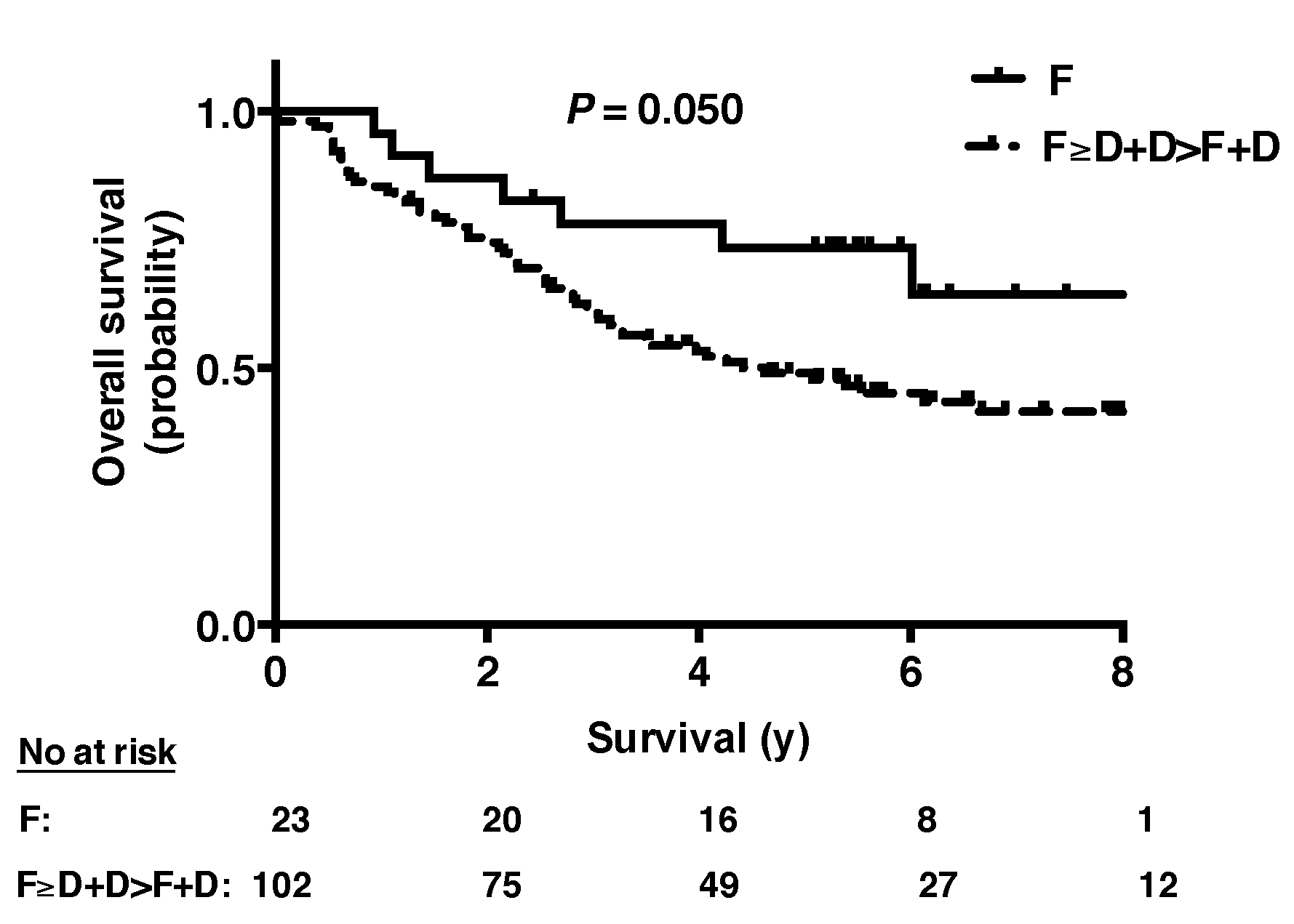

Supplement: Supplementary Fig. 1 — (JPEG 341 kb) (JPEG 316 kb) [file 259_2014_2909_Fig6_ESM.jpg]
